# Laparoscopic-assisted repair of inguinal undescended testis with concurrent incarcerated hernia in children: a single-center experience

**DOI:** 10.3389/fped.2025.1523591

**Published:** 2025-06-13

**Authors:** Jia You, Jie Sun, Shen Jing, Xin Liu, Jun Wang

**Affiliations:** ^1^ Department of Pediatric Surgery, Wuhan Children’s Hospital (Wuhan Maternal and Child Healthcare Hospital), Tongji Medical College, Huazhong University of Science and Technology, Wuhan, Hubei, China; ^2^ Department of Ultrasound, Wuhan Children’s Hospital (Wuhan Maternal and Child Healthcare Hospital), Tongji Medical College, Huazhong University of Science and Technology, Wuhan, Hubei, China

**Keywords:** laparoscopic, testis, incarcerated hernia, orchiopexy, minimal, children

## Abstract

**Purposes:**

We report clinical, operative, and outcome data for laparoscopic- assisted minimal procedure for treating unilateral inguinal undescended testis (UDT) with concurrent ipsilateral incarcerated hernia in children.

**Methods:**

Early-stage cases were defined as those presenting within 24 h of symptom onset with stable vital signs and absence of peritonitis, intestinal necrosis, or testicular necrosis. A retrospective analysis was conducted on patients undergoing laparoscopic-assisted hernia repair and trans-scrotal orchidopexy (LAHRTO) procedure.

**Results:**

A total of 14 cases were enrolled (Left, *n* = 4; Right, *n* = 10). Incarcerated hernia contents comprised viable omentum or bowel, without necrosis or intestinal perforation. Ten cases achieved successful laparoscopic reduction, while four cases required conversion to open inguinal incision due to failed reduction. All the testes were preserved and underwent the LAHRTO procedure except for those that converted. Notably, five cases of contralateral patent processus vaginalis (PPV) were identified, allowing for synchronous closure during surgery. The average operation time was (46.8 ± 5.2) min, resulting in a success rate of 71.4% (10/14). At 16–24 months of follow-up, two testes showed partial atrophy. No wound infections, hernia recurrences, or testicular retractions were observed.

**Conclusions:**

Our initial findings suggest that the LAHRTO procedure appears safe and feasible for early-stage inguinal UDT with concurrent incarcerated hernia in children, reducing inguinal incisions and enabling concurrent contralateral PPV management. A larger number of cases with longer follow-up is needed to validate the results of the current study in an evidence-based manner.

## Introduction

Undescended testis (UDT), also known as cryptorchidism, is a common congenital urogenital malformation affecting male infants and children, with a reported incidence ranging from 1% to 4% in full-term male infants and can be as high as 45% in premature (1, 2). Approximately 70% of pediatric cases of UDT involve ipsilateral hernia (3), with 12% progressing to incarceration (4). Concurrent UDT and incarcerated hernia necessitate urgent intervention to prevent ischemic sequelae.

**Figure 1:**
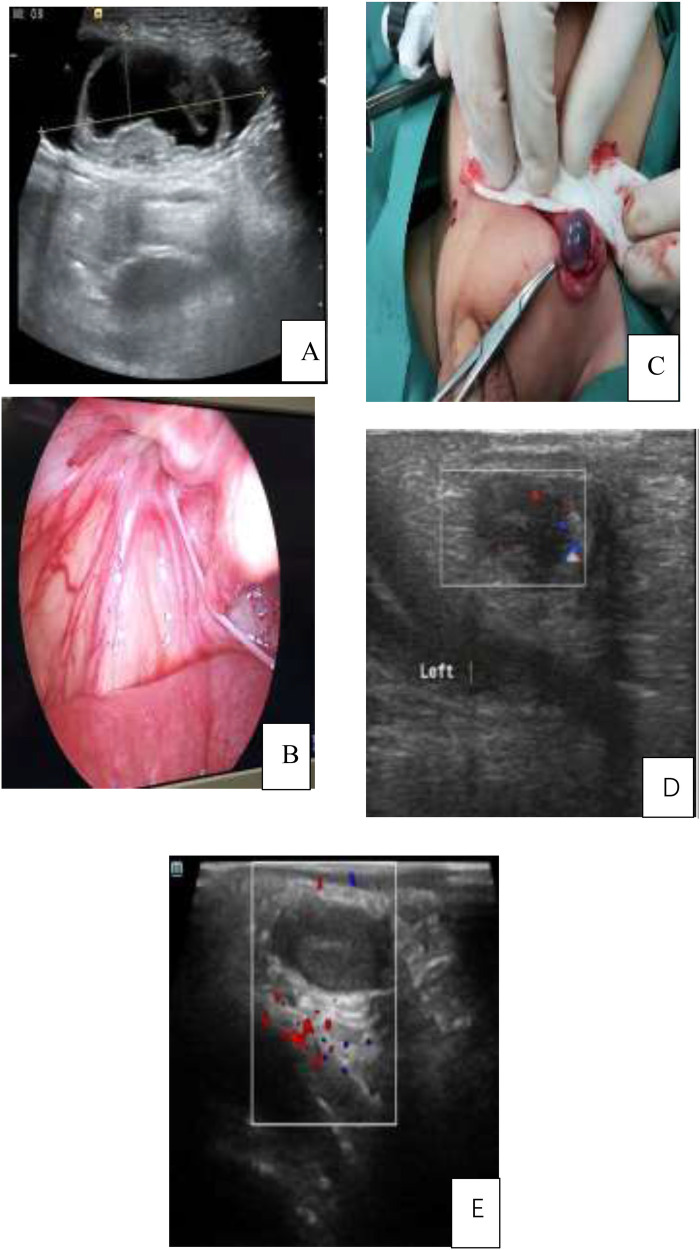
**(A)** The incarcerated contents in the left inguinal region were identified as bowel, with adjacent testicular tissue. **(B)** Successful reduction of the incarcerated bowel assisted by laparoscopy through an inguinal incision. **(C)** Intraoperative image showing partial congested testis without necrosis. **(D)** Postoperative day 3 Doppler ultrasound demonstrating testicular blood flow. **(E)** One-year follow-up ultrasound revealing partial testicular atrophy. Panel A: Ultrasound image showing a round structure with a dark center. Panel B: Endoscopic view of red tissue and vessels. Panel C: Surgical image of a gloved hand holding tissue with forceps. Panel D: Ultrasound with a rectangular overlay and colored markers. Panel E: Ultrasound with colored markers in a rectangular area.

**Table 1:** Patient characteristics of inguinal undescended testis concurrent with ipsilateral incarcerated hernia.

Case (No.)	Age (Month)	Location	Duration (Hour)	Concomitant symptom	Surgical procedure	Follow-up (Month)	Prognosis
1	19.1	Right	8	None	LAHRTO	18	Normal
2	18.1	Right	4	Vomiting	LAHRTO	24	Normal
3	17.5	Left	19	None	LAHRTO	22	Atrophy
4	16.2	Right	12	None	LAHRTO	20	Normal
5	30.9	Right	21	Vomiting	Conversion	22	Atrophy
6	13.2	Left	10	None	LAHRTO	21	Normal
7	16.6	Right	3	None	LAHRTO	18	Normal
8	14.7	Right	16	Vomiting	Conversion	19	Normal
9	45.6	Left	2	Vomiting	LAHRTO	23	Normal
10	18.1	Right	8	None	LAHRTO	21	Normal
11	18.4	Right	22	Vomiting	LAHRTO	24	Normal
12	12.7	Right	18	Vomiting	Conversion	20	Normal
13	44.8	Left	15	Nausea	Conversion	18	Normal
14	16.9	Right	6	None	LAHRTO	16	Normal

**Table 2:** Summarized data of patient characteristics.

Patient characteristics	Values
Number of patients	14
Age at surgery	Range 1–4 years
≤18 months	7
≤2 years	4
>2 years	3
Location	
Right	10
Left	4
Duration	
≤6 h	4
≤12 h	4
>12 h	6
Operation time	46.8 ± 5.2 min
LAHRTO procedure	71.4% (10/14)
Follower-up period	16–24 month
Testicular atrophy	14.3% (2/14)

Traditional open trans-inguinal approaches for UDT compromise inguinal canal integrity and prolong postoperative recovery (5, 6). Compared to the open approach, laparoscopic techniques offer comparable safety and efficacy with minimally invasive advantages. They demonstrate a superior ability to achieve optimal testicular position and success rates in the treatment of palpable UDT in children (7, 8). In recent years, the laparoscopic-assisted trans-scrotal orchiopexy technique has gained popularity for the management of inguinal UDT (9, 10). This trans-scrotal orchiopexy approach offers several advantages, including a shorter operative duration and a single aesthetic incision (11).

While research indicates that laparoscopic management of acute incarcerated hernia yields equivalent outcomes to open techniques concerning testicular atrophy and hernia recurrence (12, 13), its role in UDT with concurrent incarcerated hernia remains underexplored.

## Patient and methods

### Inclusion criteria

We define the early-stage of this condition as presentation within 24 h of onset, without peritonitis, intestinal necrosis, or testicular necrosis.

### Exclusion criteria

Patients with symptoms lasting longer than 24 h, those exhibiting intestinal perforation or signs of peritonitis, and individuals with mismatched diagnoses, such as testicular torsion of inguinal UDT, or incarcerated hernia with or without concurrent contralateral UDT, were excluded from the study.

### Surgical method

Under general anesthesia, patients were positioned in a 15°–20° Trendelenburg position. The pneumoperitoneum was established, achieving an intra-abdominal pressure of 8–12 mmHg. Standard operative techniques were employed, including the insertion and fixation of 3 mm and 5 mm trocars under laparoscopic visualization. A laparoscope and forceps were inserted through the trocar to assess intra-abdominal conditions. The team evaluated for any signs of testicular or intestinal ischemic necrosis, intestinal perforation, or the presence of a contralateral patent processus vaginalis (PPV). Hernia contents were reduced laparoscopically when viable. Subsequently, we employed a laparoscopic-assisted hernia repair and trans-scrotal orchidopexy (LAHRTO) procedure involved several key steps: patient positioning, surgical access through a transverse scrotal incision, laparoscopic visualization and management of the hernia sac, adequate mobilization, and fixation of the testis. The LAHRTO procedure was performed as described in the literature (14). If reduction was unsuccessful, a conventional surgical approach was adopted, involving an oblique inguinal incision to relieve the internal ring and restore viability; laparoscopic percutaneous extraperitoneal closure of the hernia and orchiopexy via the inguinoscrotal approach were then performed. Once the UDT was brought down into the scrotum, tension-free fixation was performed using absorbable sutures to secure the testis to the dartos fascia. In the presence of a contralateral PPV, laparoscopic extraperitoneal ligation was carried out.

## Results

### Patient demographics and preoperative findings

A total of 14 cases, aged between 1 and 4 years, were included in the study. Among these, seven instances involved children no older than 18 months, while three cases involved children older than 2 years, resulting in a median age of 17.8 months and an average age of 21.6 months, respectively. Of the cases, 10 were right-sided and 4 were left-sided. The duration of the disease varied, with four cases having a duration of ≤6 h, four cases ≤12 h, and six cases >12 h, yielding an average duration of 11.7 h. All children presented with a painful, palpable mass in the affected groin area and an absent testicle in the ipsilateral scrotum. Seven patients reported accompanying nausea or vomiting, but none exhibited signs of dehydration or fever. Preoperative color Doppler ultrasonography for all patients revealed cystic masses in the inguinal region (Figure 1A) and demonstrated testicular blood flow.

### Intraoperative observations and surgical procedure outcomes

Under microscopic observation, the peritoneum at the internal ring showed slight to moderate edema, with the incarcerated contents identified as omentum or bowel (Figure 1B). Blood flow of the intestinal segments was observed, with no signs of ischemic necrosis or intestinal perforation noted. Ten patients successfully underwent the LAHRTO procedure following reduction. The remaining four patients that required conversion to open inguinal surgery were challenging due to difficulties in achieving a reduction of the incarcerated contents (Figure 1C). In these cases, the incarcerated bowel was under significant tension, making it impossible to safely reduce laparoscopically. Intraoperatively, five cases were confirmed to have a contralateral PPV, for which synchronous closure was performed.

### Clinical and postoperative evaluation

All testes were successfully delivered into the scrotum, and an average operation time was (46.8 ± 5.2) min. Patients were discharged from postoperative care within 2–3 days. Follow-up evaluations conducted between 16 and 24 months post-surgery indicated that 12 cases demonstrated normal testicular development, while two cases exhibited partial testicular atrophy. One case involved a patient who underwent the LAHRTO procedure, and the other involved a patient whose procedure was converted to an open surgical approach (Figures 1D,E). No instances of wound infection, testicular retraction, or hernia recurrence were noted during the follow-up period. Detailed information showed in Tables 1, 2.

## Discussion

UDT is a prevalent urological condition in pediatric populations, with significant implications for male fertility and increased risks of testicular damage and malignancy if untreated (1). Current guidelines advocate orchidopexy before 18 months of age (1, 2). However, the optimal timing and surgical approach for managing palpable UDT in conjunction with an ipsilateral inguinal hernia remain debated (5, 15).

The relationship between UDT and PPV complicates management, with studies indicating that up to 90% of cases of UDT are associated with an ipsilateral PPV, leading to clinical inguinal hernias (9, 10). Prolonged abdominal pressure in these cases can contribute to hernia sac enlargement and incarceration, which occurs in approximately 12% of cases (4). The coexistence of UDT with an ipsilateral incarcerated hernia presents unique clinical challenges, including exacerbated tissue ischemia and hypoxia, and potential testicular necrosis if not addressed (16, 17).

Recent advancements in laparoscopic techniques have revolutionized the management of inguinal UDT and incarcerated hernia (9, 10, 12, 13). Studies have shown that laparoscopic-assisted trans-scrotal orchidopexy or hernia repair is a safe and effective alternative to traditional approaches, with superior outcomes in terms of preserving testicular vessels and reducing postoperative complications (14, 18–20). The minimally invasive approach offers additional benefits, including reduced tissue trauma and improved aesthetic outcomes (21, 22).

Despite these advancements, literature on the laparoscopic minimally invasive treatment for UDT coexisting with an incarcerated hernia is limited. A case reported by Marx et al. (6) in 2009 described a 24-year-old male UDT with an ipsilateral incarcerated hernia where traditional inguinal surgery was performed. More recently, Japanese researchers successfully employed laparoscopic-assisted techniques for bilateral UDT adult patient with a left incarcerated hernia, highlighting the potential for laparoscopic intervention in such cases (23).

Our findings indicate that the LAHRTO procedure is safe and effective, with a success rate of 71.4% and an average operation time was 46.8 ± 5.2 min. Patients were discharged within two to three days postoperatively. Short-term follow-up evaluations revealed that 85.7% of patients exhibited normal testicular development, although two cases experienced partial atrophy. Notably, no instances of hernia recurrence or postoperative complications, such as infection or testicular retraction, were observed. A long-term study suggests that trans-scrotal single-incision orchidopexy for palpable UDT is a safe option, with fewer than 5% requiring an additional inguinal incision, and its long-term success rate is comparable to traditional methods (21). To fully assess the long-term benefits of the LAHRTO procedure, further studies with extended follow-up periods are necessary, focusing on complications like hernia recurrence and testicular atrophy.

A key advantage of the laparoscopic approach is the ability to evaluate contralateral PPV during surgery, allowing for immediate intervention if necessary. In our study, five cases of contralateral PPV were identified, and synchronous closure was performed. All patients were under 5 years of age, and our results were consistent with those reported by Guo et al. (18) and Ma et al. (19), reinforcing the safety and effectiveness of a minimally invasive surgical approach in this age group.

Kojima et al. (24) introduced another laparoscopic approach for managing UDT with ipsilateral inguinal hernia, which involves laparoscopic-assisted trans-scrotal orchidopexy followed by laparoscopic closure of the internal ring and peritoneal defect. While this method also benefits from minimally invasive, it reported longer surgical times (89.6 ± 27.2 min), making it more suitable for non-palpable UDT or older children.

Incarcerated hernias can cause testicular ischemia in up to 56% of cases (25, 26). Our study found a testicular atrophy rate of 14.3% (2 out of 14 cases), consistent with literature rates of 6%–22% (17, 25, 26). These findings suggest that laparoscopic-assisted procedures do not increase the risk of testicular atrophy.

Despite these promising results, certain limitations warrant consideration, including the retrospective design, small sample size, and lack of a control group, which may affect the generalizability of our findings. Future multicenter studies with larger cohorts and control groups are needed for more reliable comparisons of outcomes. Additionally, challenges of the laparoscopic approach must be considered, including technical complexity, anesthesia intubation considerations, and the potential for increased operating time. Furthermore, we recommend developing an algorithm to streamline the management of UDT with incarcerated hernias, aiding clinicians in decision-making.

In conclusion, our preliminary findings suggest that the LAHRTO procedure is a feasible, effective, and minimally invasive alternative for managing inguinal UDT with an ipsilateral incarcerated hernia in young children at an early stage. This approach showcases its potential as a favorable treatment modality, although the conversion rate of nearly 30% emphasizes the need for careful patient selection and the importance of surgical expertise. Further research and longer follow-up are warranted to confirm the long-term efficacy and safety of this approach.

## Data Availability

The original contributions presented in the study are included in the article/Supplementary Material, further inquiries can be directed to the corresponding authors.
